# Towards multiomic analysis of oral mucosal pathologies

**DOI:** 10.1007/s00281-022-00982-0

**Published:** 2023-02-15

**Authors:** Jakob Einhaus, Xiaoyuan Han, Dorien Feyaerts, John Sunwoo, Brice Gaudilliere, Somayeh H. Ahmad, Nima Aghaeepour, Karl Bruckman, David Ojcius, Christian M. Schürch, Dyani K. Gaudilliere

**Affiliations:** 1 Department of Anesthesiology, Perioperative & Pain Medicine, School of Medicine, Stanford University, Stanford, CA USA; 2 Department of Pathology and Neuropathology, University Hospital and Comprehensive Cancer Center Tübingen, Tübingen, Germany; 3 Arthur A. Dugoni School of Dentistry, University of the Pacific, San Francisco, CA USA; 4 Division of Head and Neck Surgery, Department of Otolaryngology, School of Medicine, Stanford University, Stanford, CA USA; 5 Division of Plastic and Reconstructive Surgery, Department of Surgery, School of Medicine, Stanford University, 770 Welch Road, Palo Alto, CA 94304 USA

**Keywords:** Multiomics, Oral pathology, Immunology, Cytomics, Transcriptomics, Proteomics, Metabolomics, Microbiome, Mass cytometry

## Abstract

Oral mucosal pathologies comprise an array of diseases with worldwide prevalence and medical relevance. Affecting a confined space with crucial physiological and social functions, oral pathologies can be mutilating and drastically reduce quality of life. Despite their relevance, treatment for these diseases is often far from curative and remains vastly understudied. While multiple factors are involved in the pathogenesis of oral mucosal pathologies, the host’s immune system plays a major role in the development, maintenance, and resolution of these diseases. Consequently, a precise understanding of immunological mechanisms implicated in oral mucosal pathologies is critical (1) to identify accurate, mechanistic biomarkers of clinical outcomes; (2) to develop targeted immunotherapeutic strategies; and (3) to individualize prevention and treatment approaches. Here, we review key elements of the immune system’s role in oral mucosal pathologies that hold promise to overcome limitations in current diagnostic and therapeutic approaches. We emphasize recent and ongoing multiomic and single-cell approaches that enable an integrative view of these pathophysiological processes and thereby provide unifying and clinically relevant biological signatures.

## Introduction

Oral mucosal pathology is a large but understudied field that has important implications for the health and quality of life of billions of people worldwide. The most common pathologies affecting the oral mucosa fall under three categories, including (1) benign, precancerous, and malignant neoplasms (e.g., fibromas, leukoplakias, and squamous cell carcinoma), (2) bacterial, fungal, and viral infectious diseases (e.g., periodontitis, candidiasis, and herpes simplex virus), and (3) autoimmune disorders (e.g., oral lichen planus, recurrent aphthous stomatitis, pemphigus vulgaris, and mucous membrane pemphigoid). Oral pathologies affect a carefully designed barrier of the human body between a strongly bacterially colonized environment of the oral cavity and the bordering, highly vascularized and multifunctional mucosa. The mucosal integrity is crucial for central human functions such as food intake, taste, speech, breathing, and esthetics, making disability in this area truly crippling to quality of life. The localization and functionality of the mucosa as a barrier at the interface of contrasting environments imply a strong presence and involvement of the host’s immune system in the pathophysiology of most oral mucosal pathologies. An integrated examination of the immune system’s complex role in health and pathology of the oral mucosa is essential to advance the management of oral diseases.

Recently, advances in high-throughput transcriptomic, proteomic, metabolomic, and cytomic technologies have enabled the characterization of the complexity of localized and systemic diseases [1–5]. In this review, we focus on the interplay between oral pathology triggers and the host’s immune response mechanisms in the development, maintenance, progression, and resolution of pathological processes in the oral cavity. Importantly, we highlight recent technological advances that allow a multiomic assessment of immunological events involved in oral pathologies and the improvement of clinical care towards targeted immunotherapies and diagnostics.

## Immune involvement in oral pathologies

**Fig. 1:**
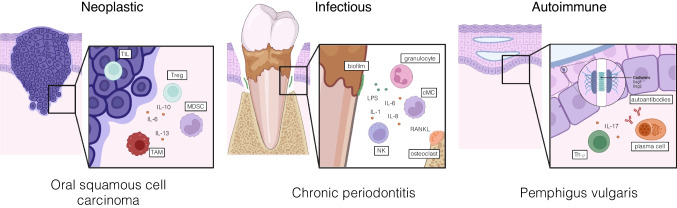
Immune involvement at the mucosal barrier. Immune cells are heavily involved in diseases of the oral mucosa. In neoplastic malignancies (*left*) such as oral cavity squamous cell carcinoma (OSCC), tumor invasion results in a pronounced immune reaction in the surrounding tissue. Abundance and composition of anti-tumor immune infiltrates in the tumor microenvironment, including tumor-associated macrophages (TAM) and tumor-infiltrating lymphocytes (TIL), as well as levels of immunosuppressive myeloid-derived suppressor cells (MDSCs) and regulatory T cells, represent prognostically relevant markers. In infectious diseases (*middle*), such as chronic periodontitis, the activation of immune cells, such as neutrophils and monocytes, by dysbiotic bacteria leads to a pronounced localized immune response resulting in the destruction of soft tissue and bone. Autoimmune diseases of the oral mucosa (*right*), such as pemphigus vulgaris, are characterized by autoreactive immune cells and autoantibodies targeting adhesive junctions in the oral mucosa. By disrupting the epithelial integrity, the inflammatory process leads to chronic blistering and painful ulcers and increases the mucosal susceptibility to bacterial infection and tissue destruction

The mucosa of the oral cavity represents an important physiological barrier of the human body and, therefore, demonstrates a high level of immune cell presence along its surface under healthy conditions. In consequence, emerging oral pathologies show a particularly strong immune involvement. The deployed functional defense mechanisms on one hand and failure, dysfunction, or hyperfunction of the host’s immune response on the other determine the development and maintenance of pathological states. In the following paragraphs, we elucidate the elemental role of immune cells in the most common neoplastic, infectious, and autoimmune diseases of the oral cavity (Fig. 1) and demonstrate how the immune system’s involvement in oral pathologies can be leveraged to enhance early detection, prevention, treatment, and resolution of these diseases.

### Neoplasms

Neoplasms or abnormal growths of tissue can occur anywhere in the body, but the oral mucosa is particularly prone to neoplastic processes, either from genetic, reactive, environmental, or unknown triggers. Oral cavity cancer, most commonly squamous cell carcinoma, accounts for approximately 2% of all cancer diagnoses [6, 7]. In many patients, oral cavity squamous cell carcinoma (OSCC) is associated with alcohol and tobacco use, a small but increasing number of cases is driven by human papilloma virus (HPV)[8], and a third category of patients have no known risk factors. Pathogenetically, the chronic insult by carcinogens leads to the development of precancerous and dysplastic lesions that develop into malignancies. Mutagenesis is also driven by chronic inflammation in the context of bacterial or viral infection [9], which is demonstrated by the strong association between persistent inflammation in chronic periodontitis and OSCC [10]. As these carcinogenic influences act on the oral cavity as a whole (field cancerization theory), cancer recurrence and metastasis, as well as synchronous or asynchronous secondary malignancies represent difficult clinical challenges and contribute to the poor prognosis (50–60% 5-year survival rate) of OSCC [11–13]. Current treatment regimes, primarily relying on surgical removal of the tumor in combination with adjuvant radiation and chemotherapy for higher tumor stages, leave room for optimization to improve outcomes, personalize treatment, and increase quality of life.

Despite many efforts to identify biological determinants of disease evolution in OSCC, existing predictive tools in patient surveillance and treatment are limited. To bridge this knowledge gap, understanding immune cell-mediated mechanisms is a high-yield approach due to the important role of the immune system in the development, progression, and metastasis of OSCC at the local and systemic levels. Locally within the tumor microenvironment, the mutual influence of innate and adaptive immune cells and cancer cells is a key determinant of tumorigenesis and the response to treatment [14, 15]. On the other hand, systemic immune dysfunction, such as immunodeficiencies and immunosuppressive treatment, increase the risk for many malignancies, including OSCC, particularly with HPV-driven pathogenesis [16, 17]. A better understanding of immune mechanisms and involvement, both detrimental and beneficial, in the pathogenesis of OSCC can be leveraged to improve early detection, prediction of outcomes, and treatment optimization strategies.

For instance, a proteomic analysis of saliva comparing patients with OSCC to healthy individuals identified significant differences in the abundance of molecules related to the acute inflammatory response and regulation of humoral immune responses (e.g., complement factors, serotransferrin, and fibrinogen) [18]. These salivary diagnostics can be useful in improving early detection protocols, as well as for tumor surveillance after initial treatment. For example, towards early detection and risk stratification, antibody positivity against HPV16 oncogenic proteins E6/E7 in the plasma years before diagnosis is highly associated with the development of HPV-positive OSCC [19]. For tumor surveillance, salivary levels of HPV DNA after surgical tumor removal have demonstrated remarkable accuracy for the prediction of OSCC recurrence [20, 21].

An analysis of the immune infiltrates at the tumor invasive front represents a promising path to predict outcomes more accurately than current tumor classification systems: expression of T cell subset markers and their distribution in and around the tumor can be useful in developing an immune scoring system that differentiates patients based on their survival [22]. In addition to the importance of histological examination of tumor tissue to quantify the immune involvement in OSCC, systemic-scale analyses reveal distinct immune changes that occur in patients with OSCC. Peripheral blood immune signatures are strongly tilted towards a state of immunosuppression, and multiple studies have found an increase in peripheral regulatory CD4^+^ T cells and myeloid-derived suppressor cells in patients with OSCC [23, 24]. These patterns of immunosuppression also recur in proteomic analyses of saliva samples that show increased concentrations of immunosuppressive IL-10 and IL-13 [25]. Suppression of anti-tumoral immune responses mechanistically should result in a worse prognosis, and indeed evidence suggests that regulatory T cells could play a role in the recurrence of OSCC [26]. However, the potential of these distinct immune signatures to predict clinical outcomes and treatment response has only been partially exploited.

As for many other cancers, immunotherapies are increasingly incorporated into clinical protocols as adjuvant or neoadjuvant treatment options. Immune checkpoint inhibitors, such as PD-1/PD-L1 or CTLA-4 inhibitors, leverage the host’s antitumoral immune response by redirecting existing immune defense mechanisms against tumor cells. Similarly, the discovery of regulatory immune receptors on tumor-fighting T cells or monocyte-derived suppressor cells, such as Vista, Tim-3, and Lag [27–29], has extended the repertoire of immunomodulatory protein targets. However, treatment success is highly variable and not all patients benefit from immune checkpoint inhibitor therapy [30]. This inconsistent treatment response may be partially explained by the fact that PD-1/PD-L1 expression is highly variable and modulated by inflammatory and hypoxic conditions in the tumor microenvironment [31, 32]. However, expression of PD-1/PD-L1 alone represents an insufficient predictor of treatment success [33]. In-depth functional and phenotypic analysis of the abundant immune populations (e.g., cytotoxic CD8^+^ T cells [34] and tumor-associated macrophages [35, 36]) at the tumor invasive front of OSCC can help identify how immune presence influences the response to immunotherapies [37, 38]. With a wider spectrum of available immunotherapies, including checkpoint inhibitors or growth-factor receptor antibodies, patient stratification approaches based on tumor phenotype and immune microenvironment are necessary to tailor the best treatment to each individual patient [39].

### Infections

Of the up to 700 species of microbes present in the oral cavity, including bacteria, fungi, viruses, and protozoa, the bacterial colonies are best characterized and have many implications for oral and systemic health. The diversity of bacterial species reflects the presence of multiple biological niches with varying conditions, from hard tissues on which the bacteria are arranged in biofilms to mucosal surfaces, from aerobic to (more pathologically) anaerobic environments (e.g., in deepening periodontal pockets under accumulated calculus) [40, 41]. This strong bacterial presence holds the pathogenetic potential for widespread diseases of the oral cavity. One of the most pertinent oral mucosal infections is periodontitis, which manifests as either a localized or generalized, acute or chronic process. In chronic periodontitis, inflammation triggered by bacteria, such as the gram-negative, facultative anaerobe *Porphyromonas gingivalis*, ultimately causes breakdown of connective tissue and alveolar bone around the teeth. Despite the initiation by bacteria, it is mainly the host’s inflammatory immune response that determines the destructive character of the disease [42]. Bacterial virulence factors, e.g., lipopolysaccharide (LPS), directly activate the host’s immune cells via Toll-like receptor (TLR) 2 and TLR4 on the surface of innate immune cells, leading to release of pro-inflammatory mediators resulting in the characteristic tissue destruction. Chronic periodontitis is highly prevalent, affecting 46% of the U.S. population [43], and it is epidemiologically associated with many other systemic conditions. In the case of cardiovascular disease, periodontal bacteria can exacerbate these conditions by translocating into the bloodstream and directly promoting the formation of atherosclerotic plaques through innate and adaptive immune mechanisms [44, 45]. Furthermore, immune cells activated locally by bacteria at the gingival sulcus circulate systemically and contribute to adverse pregnancy outcomes (preterm birth, preeclampsia), diabetes, Alzheimer’s disease, and some cancers. [46–52] To date, bacterial diseases of the mouth are often refractory to available treatments, particularly in the case of periodontitis, leading to continued insult to the mouth and body from a prolonged, infected state.

**Fig. 2:**
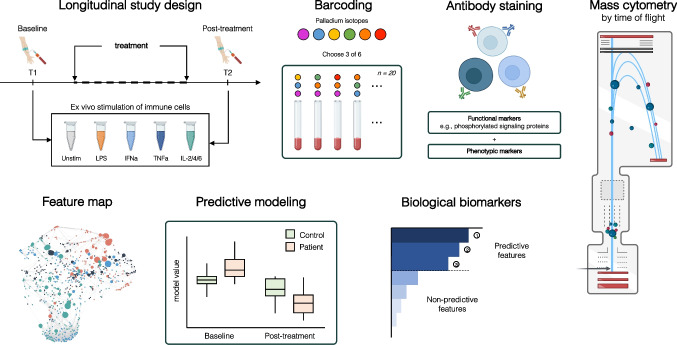
Systemic immune profiling in longitudinal studies using suspension mass cytometry (CyTOF). Using CyTOF, systemic immune signatures can be profiled in a longitudinal study design, e.g., before and after treatment. In a streamlined workflow, collected blood samples are barcoded for batch processing, stained with antibodies for phenotypic and functional markers and analyzed using CyTOF. The acquired single-cell data can be visualized and interpreted using clustering algorithms, and machine learning approaches can produce and validate reliable predictive models. In the end, the most predictive individual features are derived as biomarkers for disease, treatment success, or outcome

In chronic lesions of periodontitis, disease progression towards tissue destruction and bone loss is driven by complex interactions between periodontal bacteria and the host’s proinflammatory immune response. The release of cytokines such as receptor activator of nuclear factor kB ligand (RANK-L), interleukin (IL)-1β, IL-6, tumor necrosis factor (TNF)-ɑ, and prostaglandin E_2_a as well as increased proteolytic enzyme expression (e.g., of matrix metallopeptidase-13) by activated immune cells are immunological hallmarks of the disease [53]. Additionally, increased reactive oxygen species released by activated neutrophils and reduced antioxidative compensation, both locally and systemically, crucially contribute to periodontal pathogenesis [54]. In proteomic analyses of blood and gingival crevicular fluid, these factors can aid in the surveillance and prediction of periodontitis progression [55]. Similarly, phenotypic and functional analyses of the immune infiltrates at gingival lesions have increased our knowledge of the underlying pathomechanisms in periodontitis. Plasma cells represent one of the most predominant immune cell subsets in periodontitis and have been demonstrated to exert an important role in the initiation of osteoclastogenesis [56–58]. To determine the effectiveness of different treatment approaches, tracking the dynamic evolution of the disease over time is of crucial importance. A transcriptomic longitudinal study of periodontitis in a primate model identified gene expression patterns in the gingival tissue that demarcated phases of initiation, progression, or remission in chronic periodontitis [59]. Therefore, temporal resolution can unveil biomarkers for disease resolution or treatment success. Of particular interest are systemic immune shifts that can indicate the outcome of current, suboptimal treatment approaches, which mainly consist of scaling, root planing, and potentially local antibiotic treatment, or trials of innovative, novel therapeutics [60]. By capturing single-cell immune activation at a system level, peripheral blood signatures of active chronic periodontitis and disease remission can be recorded (Fig. 2). A recent study using suspension mass cytometry, i.e., cytometry by time-of-flight mass spectrometry (CyTOF), analysis of peripheral blood in patients with periodontitis showed heightened innate immune signaling in response to *P. gingivalis*-LPS and IL-2, 4, and 6, while adaptive immune branches showed marked inhibition of JAK/STAT signaling pathways, changes which were found to be reversible after standard treatment [61]. Such high-dimensional approaches can point towards hallmarks of localized inflammation, mechanistic links to systemic disease, and biomarkers for patient surveillance after treatment.

### Autoimmune conditions

Autoimmune diseases of the oral cavity are less prevalent than neoplastic and infectious diseases, but they cause marked reduction in quality of life, and their treatment options are often limited [62]. Oral lichen planus, recurrent aphthous stomatitis, pemphigus vulgaris, and mucous membrane pemphigoid are among the most frequently occurring autoimmune pathologies that affect the oral mucosa, and all suffer from incomplete understanding and/or lack of treatment options. Curative treatments for these diseases are often non-existent, and symptomatic management is typically achieved with blunt immunosuppressive treatments, such as topical steroids, and avoidance of exacerbating lifestyle factors, such as stress or dietary triggers [63].

One of the most common of these disorders, oral lichen planus, is a CD8^+^ T cell-mediated inflammatory condition with no known cause or cure [64, 65]. Activation of cytotoxic CD8^+^ T cell and T helper cells through antigens presented on basal keratinocytes trigger a cascade of cytokine release (e.g., TNF-α for the recruitment of other inflammatory immune cell subsets), cytotoxicity against keratinocytes (e.g., via granzyme B and Fas-ligand), and destruction of vital tissue structure (e.g., by matrix metalloproteinases) [66]. On a transcriptomic level, RNA-sequencing has allowed for identification of the dysregulated genes in oral lichen planus, which are mostly involved in T cell activation and the Wnt signaling pathway in keratinocytes [67]. Another highly prevalent disease, recurrent aphthous stomatitis, resembles oral lichen planus in its T cell-mediated inflammatory pathophysiology and episodically affects up to 20% of the population [68, 69].

Alternatively, autoreactive B cell subsets and antibody-releasing plasma cells can take center stage in autoimmune diseases: in oral pemphigus vulgaris, IgG autoantibodies are directed against members of the cadherin class of cell–cell adhesion molecules (e.g., desmoglein 1 and 3) and induce the formation of blisters by activating p38, MAPK, and mTOR signaling in keratinocytes, prompting cytoskeleton collapse and disrupting intercellular junctions [70]. Analysis of the transcriptome of pemphigus vulgaris lesions showed an IL-17A–dominated immune signature, and further analyses confirmed an increase in Th17 immune cells which contribute to the induction of desmoglein-specific autoantibody production by B cells [71, 72]. Despite the well-characterized pathophysiology of pemphigus vulgaris, corticosteroids still represent the most commonly used therapy for symptomatic management, while the development of targeted immune therapies is still in the early stages [73, 74], including promising results by targeting Bruton’s tyrosine kinase in autoreactive B cells with novel small molecule inhibitors [75]. Finally, mucous membrane pemphigoid, a similarly presenting blistering disease affecting the skin and mucous membranes, is also characterized by autoantibodies attacking epithelial structures, but the antigen targets differ from those in pemphigus vulgaris and are more variable, as they can include intracellular (BPAg1), transmembrane (BPAg2, integrins), or extracellular (collagen VII) proteins [76, 77]. Beyond the involvement of autoantibodies, little is known about the pathophysiological mechanisms of blister formation in mucous membrane pemphigoid, and targeted treatments are lacking [78].

#### High-parameter omics to identify diagnostic and therapeutic predictive biomarkers

Oral mucosal pathologies are a heterogeneous group of diseases, ranging from rare to highly prevalent conditions that have serious consequences to health and survival. For example, they can promote tumorigenesis towards the development of OSCC, exacerbate other diseases throughout the body, or cause chronic pain and functional restrictions. Despite these devastating consequences, they achieve relatively little notice in research and science. In recent years, the development of high-dimensional and single-cell technologies has enabled the assessment of cytomic, proteomic, transcriptomic, and metabolomic alterations with unprecedented resolution (Table 1). Application of these emerging omic technologies, routinely utilized to investigate other malignant, infectious, or auto-immune disease processes, is urgently needed to develop an integrative view of complex pathophysiological processes underlying oral mucosal pathologies [95].

**Table 1:**
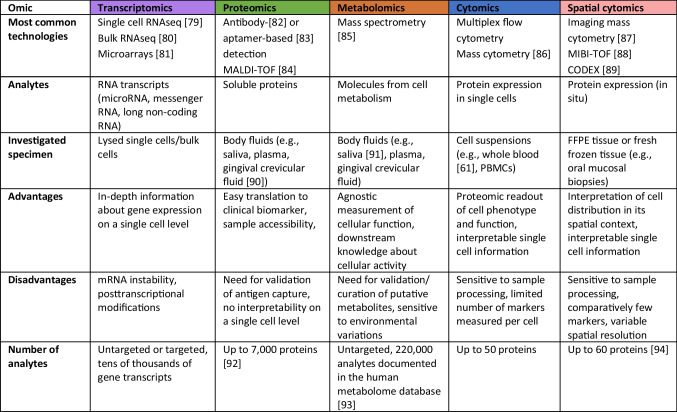
Overview: characteristics of existing omic methods. Transcriptomics, proteomics, metabolomics, and (spatial) cytomics capture biology at different levels of cellular function. While omic methods differ in their advantages and disadvantages, integrative multiomic studies can strengthen and empower the information content from each omic by describing biologically and clinically relevant interomic interconnectivity

CyTOF is a powerful analytic platform for the assessment of whole system’s immune alterations in neoplastic, infectious, and autoimmune oral pathologies. CyTOF, in contrast to conventional fluorescence-based flow cytometry, uses metal isotope-conjugated antibodies to measure over 50 parameters without significant spectral overlap on a single-cell level. Our previous work on chronic periodontitis illustrates the use of CyTOF to quantify over 800 immune cell phenotypic and functional features for an in-depth characterization of systemic immune perturbations in patients with chronic periodontitis before and after conventional treatment [61]. This longitudinal, prospective analysis identified an exaggerated proinflammatory response to *P. gingivalis*-derived LPS in neutrophils and monocytes as a main characteristic of systemic inflammation. Importantly, the differences between controls and patients with chronic periodontitis identified by a cell-signaling elastic net algorithm (csEN) markedly diminished after total-mouth disinfection treatment. In studies with larger cohorts, these findings should be tested for their generalizability, and cytomic immune profiling should be used to measure the success of new targeted treatment options.

**Fig. 3:**
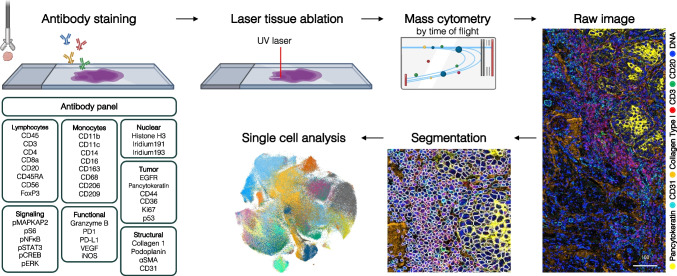
Spatial single-cell immunome studies on the horizon. Imaging mass cytometry (IMC) allows single-cell proteomics with up to 50 markers of fresh-frozen or formalin-fixed paraffin-embedded tissue slices. Pixel by pixel, the stained tissue is ablated using a UV laser and analyzed using mass spectrometry by time of flight. The raw images of marker intensities can be segmented using various cell segmentation techniques to produce single-cell data, which can be analyzed using existing bioinformatics tools for cytomic datasets

Complementing the multiplex analysis of circulating immune cells with CyTOF, high-dimensional imaging technologies have emerged and combine cell-level proteomic data with spatial information about the in situ location of single cells. Imaging mass cytometry (IMC) [87], multiplexed ion beam imaging by time of flight (MIBI-TOF) [88], or co-detection by indexing (CODEX/PhenoCycler) [89, 96] allow the simultaneous detection of up to 60 protein markers for phenotype and function in tissues. With refined deep-learning cell segmentation algorithms, raw images can be converted into single-cell data for downstream analysis that might comprise supervised manual clustering, unsupervised clustering approaches, and spatial arithmetics such as neighborhood or distance-to-border analyses (Fig. 3) [97–99]. Additionally, these platforms can enable the simultaneous detection of protein and mRNA (RNAscope) targets, as recently demonstrated by Schulz et al. for the tumor microenvironment of breast cancer and melanoma [100, 101].

While cytomic approaches (i.e., single-cell proteomics) aim to measure protein expression within individual cells, bulk proteomic assays enable the detection of soluble proteins in bodily fluids. Proteomic assays are often performed in serum or plasma allowing the identification of peripheral-blood proteomic signatures that can ultimately guide clinical decisions, for example, by differentiating between metastatic head and neck squamous cell carcinoma and primary squamous cell lung cancer [102]. However, proteomic assays are readily amenable to the analysis of other compartments, such as gingival crevicular fluid [103] or saliva [104, 105]. These oral compartments are of particular interest in studies of oral pathologies as they provide non-invasive liquid biopsies that contain biologically relevant proteomic markers, often at higher concentrations than in the circulation. Recent advances in multiplex proteomics allow the simultaneous detection of thousands of proteins using modern antibody-based (proximity extension assay, Olink) or aptamer-based (Somalogic) platforms from a small biological sample size (< 100 µl), overcoming previous constraints of limited simultaneous detection capabilities [82, 83]. The gingival crevicular fluid proteome holds important prognostic information for periodontitis progression [90], and the salivary proteome has been targeted in the search for OSCC biomarkers, yielding promising candidate biomarkers, such as elevated interleukin levels (IL-6 and IL-8), tumor antigens (CA125 and CD44), and functional proteins (Ki67 and MMP9) [106]. Hu et al. found a set of five proteomic salivary markers (M2BP, MRP14, CD59, catalase, and profilin) that identified patients with OSCC with an AUC of 93% [107]. Although limited in sample size, these studies offer promising proof-of-concept information for the use of proteomic analyses of salivary proteins to detect OSCC. Further studies will be needed to validate the findings and to integrate them with metabolomic and cytomic data.

In contrast to cytomic and proteomic platforms that provide a targeted analysis of pre-selected protein analytes, RNA sequencing (RNAseq) platforms offer agnostic detection of over 20,000 gene transcripts in bulk analysis of pooled (bulk RNAseq) or single cells (scRNAseq). A scRNAseq analysis of head and neck squamous cell carcinoma by Puram et al. described distinct transcriptional patterns in the epithelial-to-mesenchymal transition of tumor cells that are linked to nodal metastasis and histological tumor grade [108]. In other malignancies, scRNAseq not only helps elucidate key hallmarks of tumor pathogenesis and progression [109] but also optimize therapeutic strategies [110]. RNAseq approaches are also commonly utilized to characterize transcriptomic dysregulations in autoimmune diseases. In rare diseases such as pemphigus vulgaris, scRNAseq analysis can be a rewarding first approach to guide follow-up, targeted investigations. For example, a bulk RNAseq approach that was recently employed to study the dysregulated peripheral immune system of patients with pemphigus vulgaris unveiled that B cells express increased levels of IL-1β, IL-23, and IL-12 and that different treatment approaches correct these dysregulations differently [111]. Transcriptomic technologies have also evolved to enable spatial resolution of gene expression patterns in tissues. Spatial transcriptomic platforms using next-generation sequencing offer untargeted mRNA detection and can measure hundreds or thousands of genes per pixel [112, 113]. However, a technical limitation of most existing spatial RNAseq approaches is the requirement of fresh frozen tissue as these techniques are generally incompatible with formalin-fixed paraffin-embedded tissue.

In addition to proteomic and transcriptomic assays, untargeted mass spectrometry analyses of salivary metabolites have been an active field of research in the search for prognostic biomarkers in oral mucosal pathologies. In periodontitis, the metabolites cadaverine and hydrocinnamate positively correlate with the area of active inflammation, whereas uric acid and ethanolamine indicate resolution of inflammation [91]. Some of the identified metabolites in periodontitis are also predictive of OSCC suggesting a pathophysiological link and emphasizing the mutagenic potential of chronic inflammation [114–116]. Ultimately, metabolomic markers can point towards altered cellular biology and functionality in OSCC and can be used to predict overall survival [117] or response to treatment, such as chemotherapy [118].

## Integrated, multiomic modeling to identify new biological crosstalk and improve biomarker discovery

**Fig. 4:**
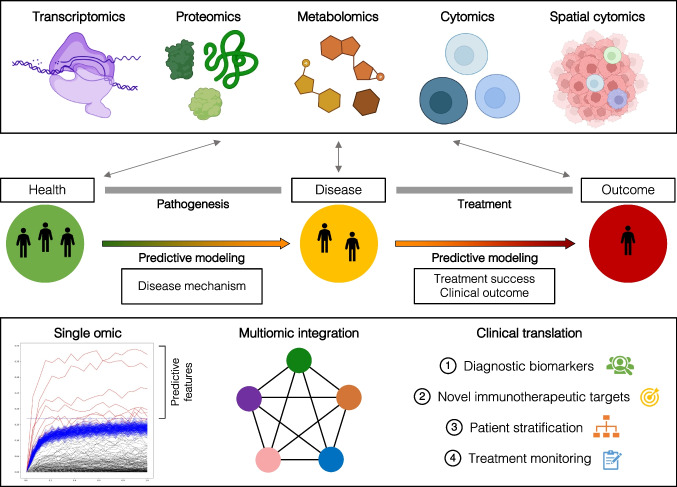
Towards integration of multiomic data layers to improve classification of oral pathologies and outcomes. Integrative studies of transcriptomic, proteomic, and cytomic biology that contribute to oral mucosal pathologies will be instrumental in defining predictive models of disease mechanism, treatment success, and outcomes. Using machine learning approaches to analyze the individual data layers and combining the predictive power of interlinked biological features will advance the discovery of diagnostic biomarkers and novel immunotherapeutic targets and enable improved patient risk stratification and treatment monitoring

Capturing multiple high-dimensional and/or single-cell modalities in a multiomic approach allows the integration of multiple biological layers into a unified biological representation of the investigated scientific question (Fig. 4). A multiomic approach also enables analysis of inter-omic crosstalk, which can be useful for confirming the validity of identified biological processes when observed in multiple data layers. However, the integration of multiple high-dimensional omic data layers poses certain statistical challenges, including differences in omic data layer dimensions, and information content. Emerging machine learning approaches have recently been developed that provide elegant solutions to concatenate multiomic information about gene expression, protein abundance, single-cell signaling activity, and phenotype as well as metabolism [119]. The timing in which the datasets are combined (early-fusion vs. late-fusion) and the applied regularization and penalization differ between analysis approaches. A commonly used approach is based on stacked generalization in which predictive models are built on each of the individual data layers before incorporating the most predictive features from each omic dataset into one overarching model [1, 3, 120]. Establishing predictive models for each data layer prior to combining them into an integrated model using stacked generalization can increase the predictive power, as this approach can account for feature intercorrelation within data layers and differences in the number of measurements between data layers. Subsequently, a post hoc correlation network analysis of selected model features can reveal relationships between features from different data layers and inform about biological cross-talks. Future challenges in computational analyses of multiomic data that are particularly relevant to complex, cross-tissue analyses of oral mucosal pathologies include the integration of spatial information such as cell–cell or cell-stroma interactions and the optimization of objective feature selection algorithms to aid in the biological interpretation of multivariate models and facilitate the biomarker discovery process.

## Conclusions

Patients with neoplastic, infectious, and autoimmune oral mucosal pathologies currently face limited and insufficient clinical treatment options. Recent proteomic, cytomic, and transcriptomic approaches provide promising avenues to elucidate mechanisms of pathogenesis and allowed for discovery of clinically relevant predictive biomarkers in distinct immune compartments. However, the implementation of single-cell and spatial omic technologies to study oral mucosal pathologies is still in its infancy. Future studies that integrate multiple omic modalities are needed to provide a comprehensive characterization of the immunological compartments that interact and contribute to disease development and resolution. Ultimately, large-scale multiomic studies in diverse patient populations will be necessary to identify and validate robust and biologically plausible signatures of clinical outcomes for the targeted development of novel (immuno)therapeutics to improve and personalize the treatment of patients with oral mucosal pathologies.

## Data Availability

Data sharing not applicable to this review article as no datasets were generated or analyzed.
